# Poly (ADP-Ribose) Polymerase Inhibitor Treatment as a Novel Therapy Attenuating Renal Ischemia-Reperfusion Injury

**DOI:** 10.3389/fimmu.2020.564288

**Published:** 2020-10-14

**Authors:** Hye Ryoun Jang, Kyungho Lee, Junseok Jeon, Jung-Ryul Kim, Jung Eun Lee, Ghee Young Kwon, Yoon-Goo Kim, Dae Joong Kim, Jae-Wook Ko, Wooseong Huh

**Affiliations:** ^1^ Division of Nephrology, Department of Medicine, Samsung Medical Center, Sungkyunkwan University School of Medicine, Seoul, South Korea; ^2^ Department of Clinical Pharmacology and Therapeutics, Samsung Medical Center, Sungkyunkwan University School of Medicine, Seoul, South Korea; ^3^ Department of Pathology, Samsung Medical Center, Sungkyunkwan University School of Medicine, Seoul, South Korea

**Keywords:** acute kidney injury, Ischemia-reperfusion injury, poly(ADP-ribose) polymerase (PARP) inhibitor, parthanatos, inflammation, translational immunology

## Abstract

Intrarenal robust inflammatory response following ischemia-reperfusion injury (IRI) is a major factor in the pathogenesis of renal injury in ischemic acute kidney injury (AKI). Although numerous studies have investigated various agents of immune modulation or suppression for ischemic AKI, few showed reproducible effects. We hypothesized that poly (ADP-ribose) polymerase (PARP) inhibitor may favorably change post-ischemic intrarenal immunologic micromilieu by reducing damage-associated molecular pattern (DAMP) signals and improve renal outcome in ischemic AKI. The effects of JPI-289 (a PARP inhibitor) on early renal injury in a murine IRI model and hypoxic HK-2 cell model were investigated. Bilateral IRI surgery was performed in three groups of 9-week-old male C57BL/6 mice (control, JPI-289 50 mg/kg, and JPI-289 100 mg/kg; n = 9–10 in each group). Saline or JPI-289 was intraperitoneally injected. Renal function deterioration was significantly attenuated in the JPI-289 treatment groups in a dose-dependent manner. Inflammatory cell infiltration and proinflammatory cytokine/chemokine expressions in the post-ischemic kidneys were also attenuated by JPI-289 treatment. JPI-289 treatment at 0.5 and 0.75 μg/ml facilitated the proliferation of hypoxic HK-2 cells. PARP inhibition with JPI-289 treatment showed favorable effects in ischemic AKI by attenuating intrarenal inflammatory cascade in a murine model and facilitating proliferation of hypoxic HK-2 cells.

## Introduction

Ischemic acute kidney injury (AKI) caused by renal ischemia-reperfusion injury (IRI) develops in various ischemic conditions of both native and transplanted kidneys. The intrarenal robust inflammatory process that is initiated and facilitated by damage-associated molecular pattern (DAMP) signals following IRI is the most crucial factor in the pathogenesis of renal injury in ischemic AKI (1, 2). Although the long-term renal outcomes of allografts have been improved in both living donor and deceased donor kidney transplantation (KT) (3, 4), the clinical impact of renal IRI is still an important unresolved issue because renal IRI causes delayed graft function (DGF) and increases risk of acute rejection in renal allografts (5, 6). Although numerous reno-protective therapies for renal IRI have been studied in animals, few have demonstrated reproducible effects in patients with ischemic AKI and none of the interventions have been translated into clinical practice (7). Therefore, the development of a novel treatment strategy for ischemic AKI is required.

Poly (ADP-ribose) polymerases (PARPs) are cell signaling enzymes that catalyze the transfer of ADP-ribose units from nicotinamide adenine dinucleotide (NAD^+^) to several acceptor proteins. PARP-1, the best characterized member of the PARP family, is an abundant nuclear enzyme implicated in the cellular response to DNA injury provoked by genotoxic stress (8). The earliest functions ascribed to PARP were DNA repair and the maintenance of genomic integrity (9). In response to low levels of DNA damage, PARP promotes cell survival and DNA repair. However, overactivation of PARP facilitates cell death through two distinct pathways: driving cells into an energetic deficit caused by depletion of intracellular NAD^+^ and catalyzing the activation of proinflammatory pathways (9, 10). This overactivation of PARP by a wide array of stimuli including reactive oxygen species (ROS), termed parthanatos, is implicated in the pathogenesis of renal IRI (7), suggesting that PARP is a potential treatment target in renal IRI. Parthanatos can be blocked by genetic deletion of PARP-1 or pharmacological inhibition of PARP-1 by PARP inhibitors. PARP-1-deficient mice were protected in renal IRI (11). JPI-289, a recently developed novel PARP-1 inhibitor with strong PARP-1 inhibitory activity, showed beneficial effects in ischemic stroke models (12, 13).

In this study, we hypothesized that pharmacologic PARP-1 inhibition with JPI-289 may favorably change the postischemic intrarenal immunologic micromilieu and mitigate early renal injury following IRI.

## Materials and Methods

### Study Design

We used an established murine IRI model for *in vivo* study and HK-2 cell (an immortalized proximal tubule epithelial cell line from normal adult human kidney) hypoxia model for *in vitro* study to investigate the effects of JPI-289 on ischemic AKI. In the murine IRI model, measurements of plasma creatinine and blood urea nitrogen (BUN) concentration and histological assessment of renal tissues were performed to assess functional and structural renal outcomes. For comprehensive analysis of the immunologic mechanism, intrarenal leukocyte trafficking, phenotypes of kidney mononuclear cells (KMNCs), and the expressions of intrarenal cytokines/chemokines and proinflammatory signaling pathways were assessed in post-ischemic kidney tissues. Cell proliferation and the expression of inflammatory/apoptotic signaling molecules were measured in the HK-2 cell hypoxia model. We also measured PARP-1 activities in the dose of JPI-289 applied in the hypoxic HK-2 cell model with biotinylated poly (ADP-ribose) loaded samples and the mice postischemic kidney protein extracts to test the PARP-1 suppression activity of JPI-289.

### Mice and Renal IRI Model

The Samsung Medical Center Animal Care and Use Committee approved all studies. This study was approved by the institutional review board of Samsung Medical Center. Animal studies are reported in compliance with the ARRIVE guidelines (14, 15). Male C57BL/6 mice (9 weeks old) were purchased from Orient Bio Inc. (Seongnam, Kyoungki-do, Korea). All mice were housed in a specific pathogen-free barrier facility.

Mice were anesthetized with an intraperitoneal injection of ketamine (100 mg/kg; Yuhan, Seoul, Korea) and xylazine (10 mg/kg; Bayer, Leverkusen, Germany). After an abdominal midline incision, the renal pedicles of both kidneys were carefully isolated and clamped for 27 min with a microvascular clamp (Roboz Surgical Instrument, Gaithersburg, MD). During the surgery, anesthetized mice were placed onto a thermostatically controlled heating table and kept well hydrated with warm sterile saline. After 27 min, microvascular clamps were released from renal pedicles for reperfusion. After being sutured, mice were allowed to recover with free access to chow and water. Sham controls underwent the same surgical procedure except for clamping of the renal pedicles. All mice were sacrificed on day 3 after surgery and kidneys were harvested after exsanguination.

### Administration of JPI-289

Renal IRI model mice were randomized into three groups: control (n = 10), JPI-289 50 mg/kg (n = 10), and JPI-289 100 mg/kg (n = 9). JPI-289, 10-ethoxy-8-(morpholinomethyl)-1,2,3,4-tetrahydrobenzo [h] [1,6] naphtyridin-5(6H)-one dihydrochloride dehydrate, was synthesized by Jeil Pharmaceutical Co. Ltd (Seoul, Korea). JPI-289 (50 mg/kg or 100 mg/kg) or saline were administered twice intraperitoneally: right before reperfusion during the IRI surgery and at 24 h after the surgery. The surgery and administration of JPI-289 were performed by different investigators than the following experiments, ensuring that all analyses were performed in a blinded fashion.

### Assessment of Renal Function

Plasma creatinine (Arbor Assays, Ann Arbor, MI) and BUN (Fujifilm, Bedford, UK) levels were measured in plasma samples obtained on days 1, 2, and 3 after surgery using colorimetric kits according to the manufacturer’s recommended methods. Baseline plasma creatinine and BUN levels were measured at 7 days before surgery.

### Measurement of PARP-1 Activity

The PARP-1 inhibitory effect of JPI-289 was assessed using a colorimetric PARP assay kit (R&D Systems, Minneapolis, MN) based on the incorporation of biotinylated ADP-ribose into histone proteins. Briefly, samples were loaded onto a 96-well plate coated with histones and biotinylated poly (ADP-ribose), incubated for 30 min followed by strep-HRP treatment, and read at 450 nm on a spectrophotometer.

### Tissue Histological Analysis

After exsanguination, both postischemic kidneys were harvested. Tissue sections were fixed with 10% buffered formalin followed by paraffin embedding and staining with hematoxylin and eosin. A renal pathologist who was blinded to the experimental groups scored renal tubular damage of postischemic kidneys and the percentage of necrotic tubules were compared.

### Assessment of Leukocyte Phenotype

KMNCs were isolated according to previously described methods (16). Briefly, decapsulated kidneys were immersed in RPMI buffer (Mediatech, Manassas, VA) containing 5% FBS and disrupted mechanically using a Stomacher 80 Biosmaster (Sweward, Worthing, West Sussex, UK). Samples were strained, washed, and resuspended in 36% Percoll (Amersham Pharmacia Biotech, Piscataway, NJ) followed by gentle overlaying onto 72% Percoll. After centrifugation at 1,000 g for 30 min at room temperature, KMNCs were collected from the interface of 36% and 72% Percoll. KMNCs were then washed twice and counted on a hemocytometer using trypan blue exclusion.

Isolated KMNCs were preincubated with anti-CD16/CD32 Fc receptor blocking antibody for 10 min to minimize nonspecific antibody binding. KMNCs were then incubated with anti-mouse anti-CD3, -CD4, -CD8, -CD19, -CD21/35, -CD25, -CD27, -CD44, -CD45, -CD62L, -CD69, -CD126, -CD138, -F4/80, -FoxP3, -GR1, -TCRβ, and -NK1.1 (all from BD Biosciences, San Jose, CA) for 25 min at 4°C, washed with FACS buffer, and fixed with 1% paraformaldehyde solution. Acquisition and analyses of eight-color immunofluorescence staining were performed using a FACSVerse instrument and FACSuite program (BD Biosciences), respectively. Each assay included at least 10,000 gated events. Detailed gating strategies for flow cytometry analyses are provided in **Supplemental Figures**.

### Immunohistochemistry of Renal Tissues With CD45

Formalin-fixed renal tissue sections were stained for CD45 using immunohistochemistry. Sections (4-µm-thick) were deparaffinized with xylene, rehydrated in a graded alcohol series, and then transferred to citrate buffer solution (pH 6.0). Slides were placed in a pressure cooker and heated by microwaving for 10 min to enhance antigen retrieval. After cooling, the kidney sections were immersed in a hydrogen peroxide solution (DAKO, Carpinteria, CA) for 30 min to block endogenous peroxidase activity, followed by overnight incubation at 4°C with serum-free protein block (DAKO). The next day, the slides were incubated with a 1:100 dilution of anti-mouse CD45 monoclonal antibody (BD Biosciences, San Jose, CA) for 1 h at room temperature. After being rinsed, the CD45-stained sections were incubated for 30 min at room temperature with a secondary antibody using a Dako REAL EnVison kit (DAKO). Subsequently, 3,3’-diaminobenzidine tetrahydrochloride (DAKO) was applied to the slides to produce a brown color and then the slides were counterstained with Mayer’s hematoxylin solution (DAKO).

To calculate the percentage of CD45-positive cells in kidney samples, whole fields of slides including both cortex and medulla were scanned and analyzed with a TissueFAXS work station (Tissue Gnostics, Vienna, Austria), as described previously (17).

### Multiplex Cytokine/Chemokine Assay

A panel of cytokines and chemokines was measured in whole kidney protein extracts using the Milliplex MAP Mouse Cytokine/Chemokine Kit (Luminex, Austin, TX) according to the manufacturer’s instructions. This multiplexed, particle-based, flow cytometric assay uses anti-cytokine monoclonal antibodies linked to microspheres incorporating distinct properties of two fluorescent dyes. Our assay was designed to quantify interleukin (IL)-2, IL-4, IL-6, IL-10, interferon (IFN)-γ, CC-chemokine ligand (CCL)-2 (monocyte chemoattractant protein-1), CCL5 (regulated on activation, normal T cell expressed and secreted), tumor necrosis factor (TNF)-α, and vascular endothelial growth factor (VEGF). The limit of detection values of each cytokine/chemokine were as follows: IL-2, 1.0 pg/ml; IL-4, 0.4 pg/ml; IL-6, 1.1 pg/ml; IL-10, 0.8 pg/ml; IFN-γ, 1.1 pg/ml; CCL-2, 6.7 pg/ml; CCL-5, 2.7 pg/ml; TNF-α, 2.3 pg/ml; VEGF 0.3 pg/ml. The concentration (pg/ml) of intrarenal cytokine or chemokine was normalized by dividing the raw protein concentration (mg/ml, measured by Pierce BCA protein assay kit, Thermo Fisher Scientific, Waltham, MA) of whole kidney protein extract. Therefore, the final unit of cytokine/chemokine value was expressed as “pg/mg”.

### Western Blot Analysis

Toll-like receptor (TLR)-4, nuclear factor kappa-light-chain-enhancer of activated B cells (NFκB), Bax, and Bcl-2 were assayed by western blot analysis. Equal amounts of whole kidney protein extract (30 µg) were separated by electrophoresis on a NuPAGE Bolt mini gel system (Thermo Fisher Scientific) following the manufacturer’s instructions. After electrophoresis, the gels were transferred onto a nitrocellulose membrane using an iBot 2 Dry Bottling system (Thermo Fisher Scientific). Membranes were blocked with 5% skim milk tris-buffered saline solution with 0.1% Tween20 for 1 h at room temperature and then incubated overnight at 4°C with one of the following antibodies: mouse monoclonal anti-TLR4 antibody (Novus Biologicals, Centennial, CO), mouse monoclonal anti-NFκB antibody (R&D Systems), rabbit anti-Bax antibody (Cell Signaling Technology Inc., Danvers, MA), rabbit anti-Bcl-2 antibody (Cell Signaling Technology Inc.). After washing with TBST, the horseradish peroxidase-conjugated secondary antibody was applied for 30 min at room temperature. After washing, the signal was visualized using the Amersham ECL detection system (GE Healthcare, Chicago, IL), according to the manufacturer’s instructions. Bands were then densitometrically analyzed using ImageJ 1.52k software (Wayne Rasband, National Institutes of Health, MD). Band density, expressed as an arbitrary unit (AU), was normalized against corresponding β-actin band intensities as an internal control.

### HK-2 Cell Hypoxia Model

HK-2 cells were purchased from American Type Culture Collection (CRL-2190, Manassas, VA) and cultured in keratinocyte serum-free media (Thermo Fisher Scientific) supplemented with bovine pituitary extract and human recombinant epidermal growth factor. Cells were incubated at 37°C in humidified atmosphere of 5% CO_2_ with media changes every 2 to 3 days.

Hypoxia was induced by exposure to 1% O_2_ and 5% CO_2_ balanced with nitrogen in a multi-gas incubator (APM-30D, Astec, Fukuoka, Japan) for 48 h. HK-2 cells were divided into 11 groups. The 2 control groups were the normoxia control group (21% O_2_) and the hypoxia control group (1% O_2_). The single-dose groups were treated with 0.1, 0.25, 0.5, 0.75, or 1.0 µg/ml of JPI-289 on day 0 (the day when cells were taken out from the multi-gas incubator) after hypoxia. The double-dose groups were treated with 0.1, 0.25, 0.5, and 0.75 µg/ml of JPI-289 on day 0 and day 1 after hypoxia.

HK-2 cells under normoxia were also treated with above mentioned dosages of JPI-289 to explore the effect of JPI-289 on normal kidney.

The degree of HK-2 cell proliferation on day 0, day 1, and day 2 was assessed with the Cell Titer96 aqueous one solution cell proliferation assay (Promega, Madison, WI) according to the manufacturer’s instructions.

To further analyze the effect of JPI-289 on hypoxia-induced cell signaling pathways and apoptosis, TLR-4 and NFκB were measured with western blot, and Bax and Bcl-2 were measured with an enzyme-linked immunosorbent assay (ELISA) kit (Abcam, Cambridge, MA).

### Data and Statistical Analyses

All data were expressed as mean ± standard error of mean (SEM). Group means were compared with Mann-Whitney *U* test, Kruskal-Wallis test followed by Dunn’s test, or ANOVA followed by Newman-Keuls *post hoc* analysis using GraphPad Prism version 8 (GraphPad Software, La Jolla, CA). *P* values < 0.05 were considered statistically significant.

## Results

### JPI-289 Treatment Mitigated Functional and Structural Renal Injury Following IRI

**Figure 1:**
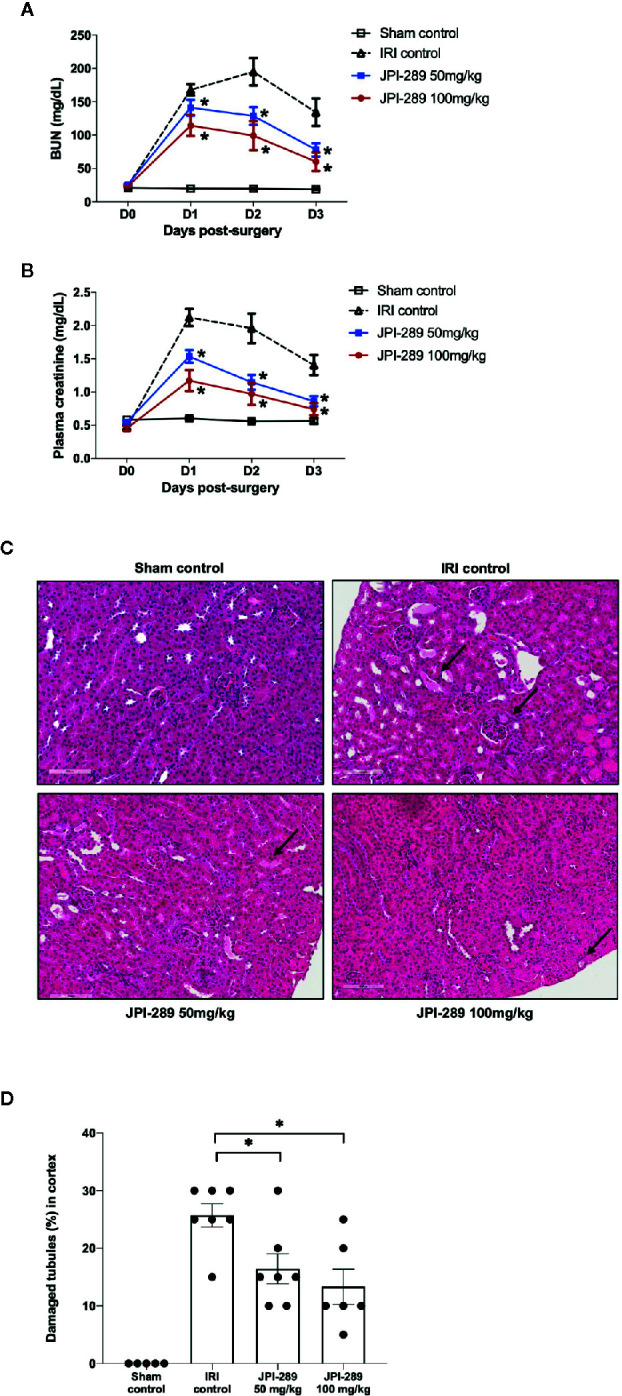
Functional and structural renal injury following IRI. **(A, B)** Blood urea nitrogen (BUN) and plasma creatinine levels were significantly lower in the JPI-289-treated groups compared with the IRI control group in a dose-dependent manner. **(C)** Hematoxylin and eosin staining of the cortex of postischemic kidneys. Arrows indicate damaged or necrotic tubules (×200). **(D)** On day 3 after IRI, the JPI-289-treated groups showed less damaged or necrotic tubules compared to the IRI control group. A total of 10 fields magnified 200× were scored for each mouse by a pathologist blinded to the groups. Data are from three independent experiments. *P < 0.05, compared with the IRI control group (*n* = 6–10 in each IRI group, *n* = 5 in the sham control group). Statistical analyses were performed with two-way ANOVA test followed by Newman-Keuls test **(A, B)** or the Mann-Whitney *U* test **(D)**.

Renal function following the operation was evaluated with daily measurement of BUN and plasma creatinine up to day 3 after the operation. BUN and plasma creatinine were stable in the sham control group but increased following IRI. Both BUN and plasma creatinine were significantly lower in the JPI-289–treated groups compared with the IRI control group in a dose-dependent manner (**Figures 1A, B** **)**. We evaluated the tubular injury of cortex using H&E staining. Postischemic kidneys of the IRI model mice showed marked tubular damage compared to the sham control group on day 3 after operation. The proportion of damaged or necrotic tubules was significantly lower in the cortex of the JPI-289–treated groups compared with the IRI control group (**Figures 1C, D** **)**.

### JPI-289 Treatment Reduced Leukocyte Trafficking into Postischemic Kidneys

**Figure 2:**
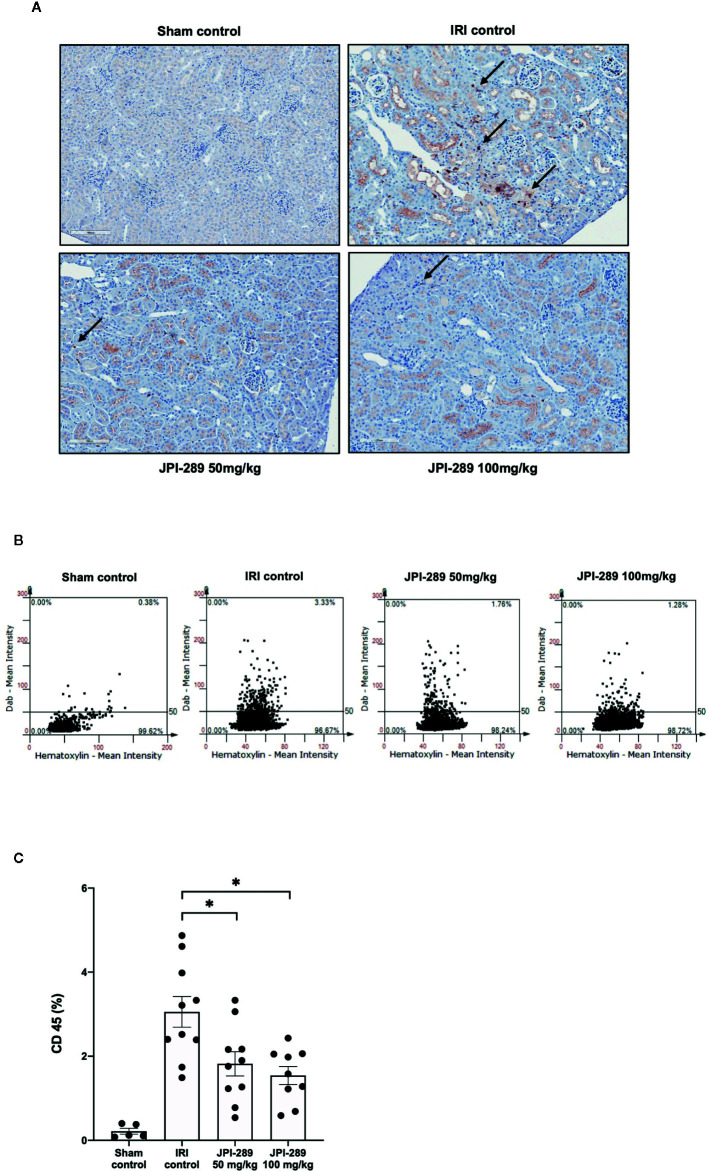
Leukocyte trafficking into the postischemic kidneys. **(A)** There were more pronounced infiltrations of leukocytes into the postischemic kidneys of IRI control mice compared with JPI-289-treated groups. Arrows indicate CD45-positive leukocytes (×200). **(B)** Semiquantitative analysis of CD45-positive leukocytes in the postischemic kidney using automated imaging analysis system (TissueFAXS). The whole fields of slides including both cortex and medulla were evaluated. **(C)** The percentages of total leukocytes expressing CD45 among total nucleated cells were lower in the postischemic kidneys of JPI-289-treated mice compared with those of IRI control mice. Data are from three independent experiments. *P < 0.05, compared with the IRI control group (*n* = 6–10 in each IRI group, *n* = 5 in the sham control group). Statistical analysis was performed using the Mann-Whitney *U* test.

The trafficking of total leukocytes into postischemic kidneys was evaluated using immunohistochemical staining of CD45. The proportion of total leukocyte expressing CD45 among total nuclei in the whole field of each slide was semiquantitatively calculated with TissueFAXS system. Intrarenal total leukocytes expressing CD45 significantly increased in the postischemic kidneys compared to the kidneys of sham control mice. The percentage of total leukocytes was significantly lower in the JPI-289 treatment groups in a dose-dependent manner (CD45-positive cells among total nucleated cells, sham control vs. IRI control vs. 50 mg/kg vs. 100 mg/kg of JPI-289 (mean ± SEM): 0.22 ± 0.07 vs. 3.05 ± 0.37 vs. 1.82 ± 0.29 vs. 1.54 ± 0.21; **Figure 2**).

### JPI-289 Treatment Decreased Intrarenal NK Cells and Macrophages

**Figure 3:**
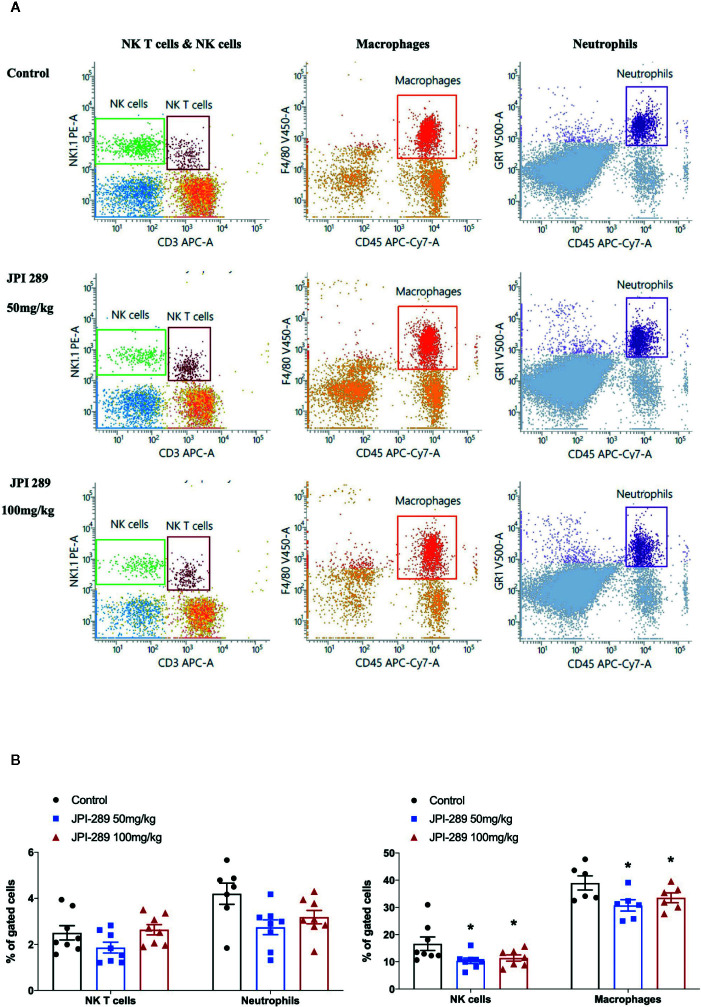
Flow cytometry analyses of KMNCs isolated from postischemic kidneys on day 3 after IRI. **(A, B)** The infiltration of NK T cells were comparable among the 3 groups. The infiltration of neutrophils tended to be lower in the JPI-289 treated group. JPI-289 reduced the infiltration of intrarenal NK cells and macrophages. Data are from two independent experiments. *P < 0.05, compared with the control group (*n* = 6–8 in each group). Statistical analysis was performed with the Kruskal-Wallis test followed by Dunn’s test. Detailed gating strategies for each population were as follows; Lymphocytes, monocytes, and granulocytes were first identified on the basis of their FSC and SSC. CD45^+^ cells were gated to identify lymphocytes within the FSC and SSC based lymphocytes population. NK T cells were identified by CD3^+^ and NK1.1^+^ gate within lymphocytes. NK cells were identified by CD3^-^ and NK1.1^+^ gate within lymphocytes. Macrophages were identified by CD45^+^ and F4/80^+^ gate within the FSC and SSC based monocyte population. Neutrophils were identified by CD45^+^ and GR1^+^ gate within FSC and SSC based granulocyte population (more details are provided in **Supplemental Figure 3**).

We analyzed the phenotypes of KMNCs by flow cytometry to assess the effect of JPI-289 on major effector cells of both innate and adaptive immune systems in the postischemic kidneys on day 3 after IRI. The percentages of total T cells, CD4 and CD8 T cells, activated CD4 and CD8 T cells, regulatory T cells, and effector memory CD4 and CD8 T cells were comparable between the control group and the JPI-289 treatment groups (**Table 1**). The percentages of total B cells, subpopulations of B cells, NK T cells were also comparable between groups. Although JPI-289–treated groups showed lower percentages of neutrophils, the differences were not statistically significant. However, intrarenal NK cells and macrophages on day 3 after IRI surgery were decreased by JPI-289 treatment (**Figure 3**).

**Table 1:** The leukocyte populations in the postischemic kidney on day 3 after IRI.

Leukocytes’ types (% in parent gate)	Control	JPI-289 50 mg/kg	JPI-289 100 mg/kg
Of size and granularity-based gate			
Macrophages, % NK T cells, % NK cells, %	39.0 ± 2.6 2.5 ± 0.3 16.7 ± 2.5	30.8 ± 2.1* 1.9 ± 0.2 10.4 ± 1.0*	33.9 ± 2.2* 2.5 ± 0.2 11.4 ± 1.4*
Of size and granularity-based gate			
Neutrophils, %	4.9 ± 0.8	2.7 ± 0.3	3.2 ± 0.3
Among total lymphocytes			
Total T cells, %	25.1 ± 1.7	23.7 ± 1.1	22.3 ± 2.4
Total B cells, %	37.1 ± 2.5	42.1 ± 4.2	42.3 ± 1.9
Among total T cells			
CD4 T cells, %	67.9 ± 2.1	63.6 ± 1.6	65.8 ± 2.0
CD8 T cells, %	12.7 ± 1.6	15.1 ± 1.1	13.0 ± 1.7
Among total CD4 T cells			
Activated CD4 T cells, %	3.2 ± 0.3	3.2 ± 0.4	3.7 ± 0.3
Effector memory CD4 T cells, %	39.5 ± 2.9	39.6 ± 3.6	42.8 ± 4.9
Regulatory T cells, %	0.7 ± 0.1	0.5 ± 0.1	0.8 ± 0.2
Among total CD8 T cells			
Activated CD8 T cells, %	2.3 ± 0.6	2.5 ± 0.4	2.1 ± 0.6
Effector memory CD8 T cells, %	14.9 ± 2.6	15.2 ± 3.2	20.3 ± 5.2
Among total B cells			
Activated B cells, %	1.1 ± 0.2	1.1 ± 0.2	1.1 ± 0.1
Mature B cells, %	93.3 ± 0.7	93.5 ± 1.2	94.6 ± 0.5
Memory B cells, %	5.1 ± 0.2	5.5 ± 0.7	6.1 ± 0.4
Plasma cells among KMNCs	0.3 ± 0.1	0.2 ± 0.1	0.3 ± 0.1

Data (mean ± standard error of mean) are from two independent experiments. *P < 0.05, compared with the control group (n = 6–8 mice in each group).

Detailed gating strategies are provided in **Supplemental Materials**.

IRI, ischemia-reperfusion injury; KMNCs, kidney mononuclear cells.

### JPI-289 Treatment Altered Cytokine and Chemokine Expressions in Postischemic Kidneys

**Figure 4:**
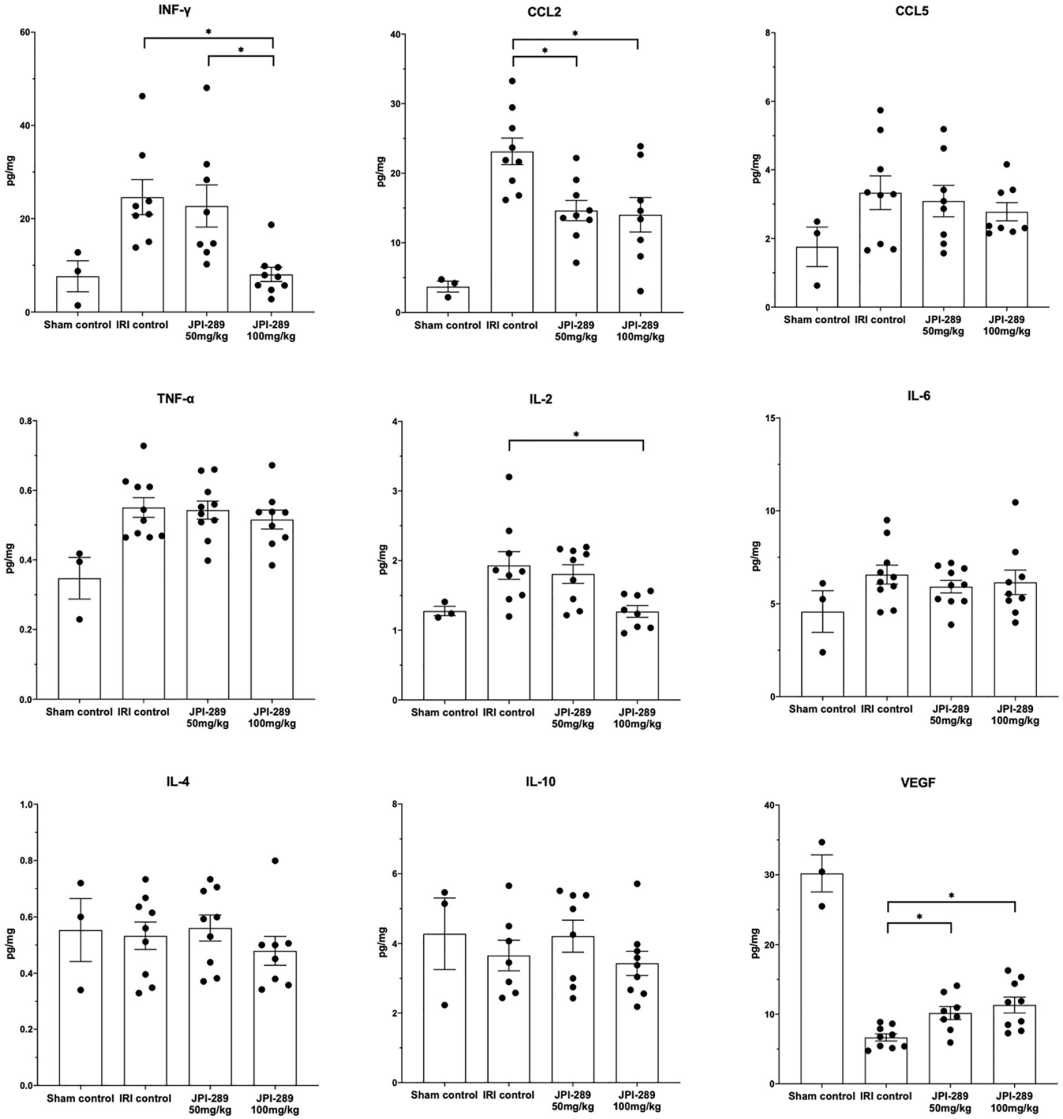
The expression of intrarenal cytokines and chemokines in the postischemic kidneys on day 3 after IRI. JPI-289 treatment significantly decreased the expression of IFN-γ, CCL2, and IL-2 and increased the intrarenal expression of VEGF. *P < 0.05, compared with the IRI control group (*n* = 6–10 in each IRI group, *n* = 3 in the sham control group). Kidney protein extracts were obtained from three independent experiments. Statistical analysis was performed with the Kruskal-Wallis test followed by Dunn’s test.

To explore whether JPI-289 treatment affects intrarenal expressions of major pro- and anti-inflammatory cytokines/chemokines in postischemic kidneys, we analyzed intrarenal expression of IFN-γ, CCL2, CCL5, TNF-α, IL-2, IL-4, IL-6, IL-10, and VEGF. Comparison of the sham control group and the IRI control group showed that IRI enhanced intrarenal expressions of IFN-γ (*P* = 0.012), CCL2 (*P* = 0.009), TNF-α (*P* = 0.007), and IL2 (*P* = 0.036), and suppressed the expression of VEGF (*P* = 0.009). The expressions of proinflammatory cytokines/chemokines including IFN-γ, CCL2, and IL-2 were significantly lower in the JPI-289 treatment groups compared with the IRI controls. In contrast, the intrarenal expression of VEGF was significantly higher in the JPI-289 treatment groups compared with the IRI control group. The expressions of CCL5, TNF-α, IL-4, IL-6, and IL-10 were comparable between IRI groups (**Figure 4**).

### JPI-289 Treatment Suppressed TLR4 and NFκB Expression in Postischemic Kidneys

**Figure 5:**
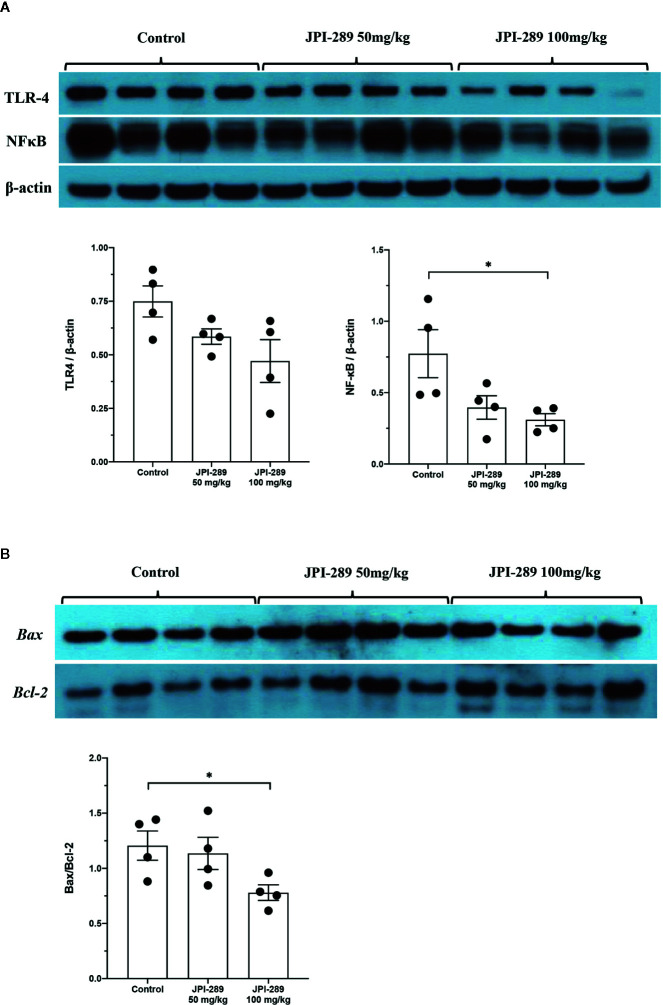
Western blotting of protein samples extracted from postischemic kidneys and densitometry analyses. **(A)** The expression of NFκB was significantly decreased by JPI-289 treatment. The expression of TLR4 tended to be lower with JPI-289 treatment. **(B)** Bax/Bcl-2 ratios were lower in the JPI-289 treatment groups. *P < 0.05, compared with the control group. Kidney protein extracts (*n* = 6–10 in each group) were obtained from two independent experiments. Statistical analysis was performed with the Mann-Whitney *U*-test.

Since TLR4 and NFκB pathways are known to play critical roles in renal IRI by activating series of inflammatory genes and innate immune response, intrarenal expressions of TLR4 and NFκB were evaluated by western blotting of protein samples extracted from postischemic kidneys. The expressions of TLR4 tend to be lower with JPI-289 treatment. The expression of NFκB in the JPI-289 100 mg/kg group was significantly lower compared with the control group (**Figure 5A**).

### JPI-289 Inhibited Apoptosis in Postischemic Kidneys

As apoptosis is a central feature of renal IRI, we measured the expression of an antiapoptotic molecule, Bcl-2, and proapoptotic molecule, Bax to examine the molecular responses associated with apoptosis pathway. Western blot assays of Bcl-2 and Bax proteins showed that JPI-289 downregulated the expression of Bax and upregulated the expression of Bcl-2 in the JPI-289 100 mg/kg group compared with controls (*P* = 0.03 between control group and 100 mg/kg of JPI-289 group, **Figure 5B**).

### JPI-289 Treatment Facilitated the Proliferation of Hypoxic HK-2 Cells

**Figure 6:**
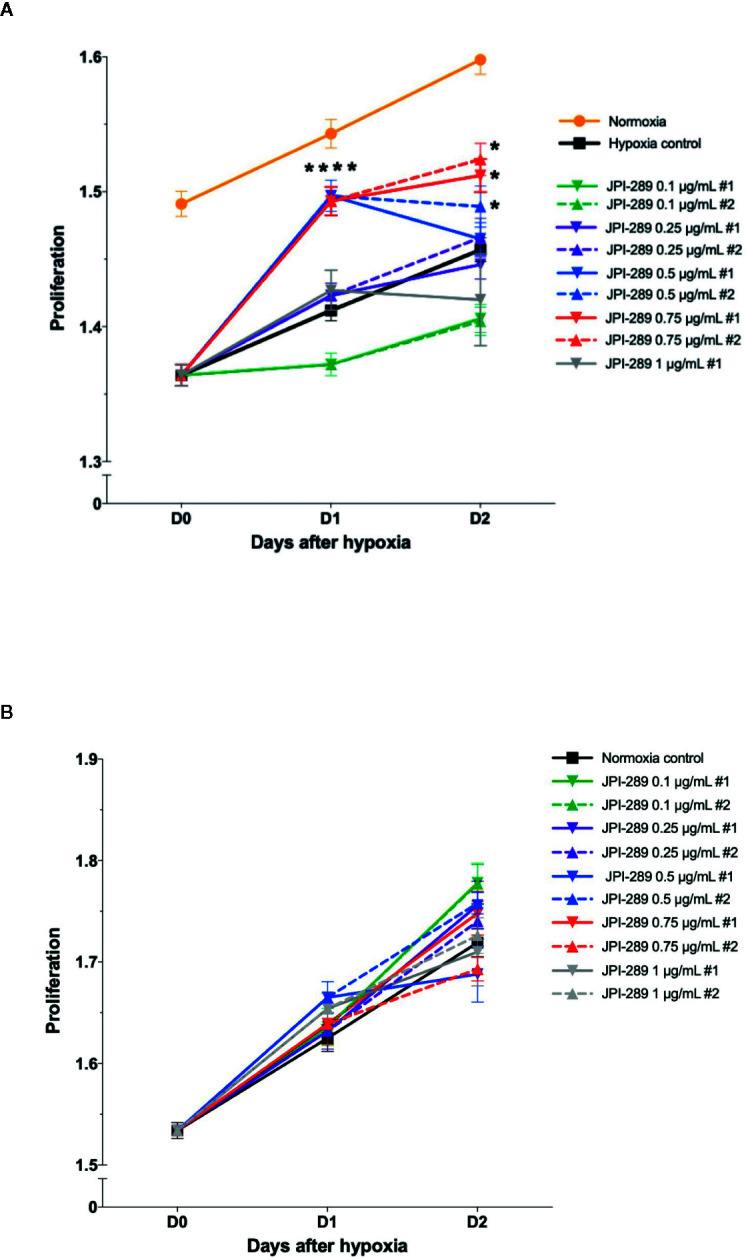
Proliferation of hypoxic HK-2 cells according to the dose of JPI-289. **(A)** JPI-289 treatment at 0.5 or 0.75 µg/ml facilitated proliferation of hypoxic HK-2 cells compared with the hypoxia control group. Day 0 corresponds to the day when HK-2 cells were taken out from the multi-gas incubator after 48 h of hypoxia. Data are from eight independent experiments. *P < 0.05, compared with the hypoxia control group. Statistical analysis was performed with the Mann-Whitney *U* test. **(B)** The proliferation of normoxic HK-2 cells treated with JPI-289 was comparable with that of the normoxia control group. Data are from eight independent experiments.

To explore the effects of JPI-289 on human postischemic kidney, hypoxic HK-2 cells were treated with JPI-289. **Figure 6A** shows the degree of HK-2 cell proliferation after hypoxic insult according to the dose of JPI-289. Following hypoxic insult, 0.5 or 0.75 µg/ml of JPI-289 treatment facilitated the proliferation of hypoxic HK-2 cells compared with the hypoxia control group. However, lower or higher dosages, such as 0.25 or 1 µg/ml of JPI-289, did not show favorable effects on hypoxic HK-2 cells, which suggest that appropriate dosage may be important for attenuating IRI (**Figure 6A**). Under normoxic condition, JPI-289 treatment did not affect the proliferation of HK-2 cells (**Figure 6B**).

### JPI-289 Treatment Suppressed TLR4 and NFκB Signaling Pathway in Hypoxic HK-2 Cells

**Figure 7:**
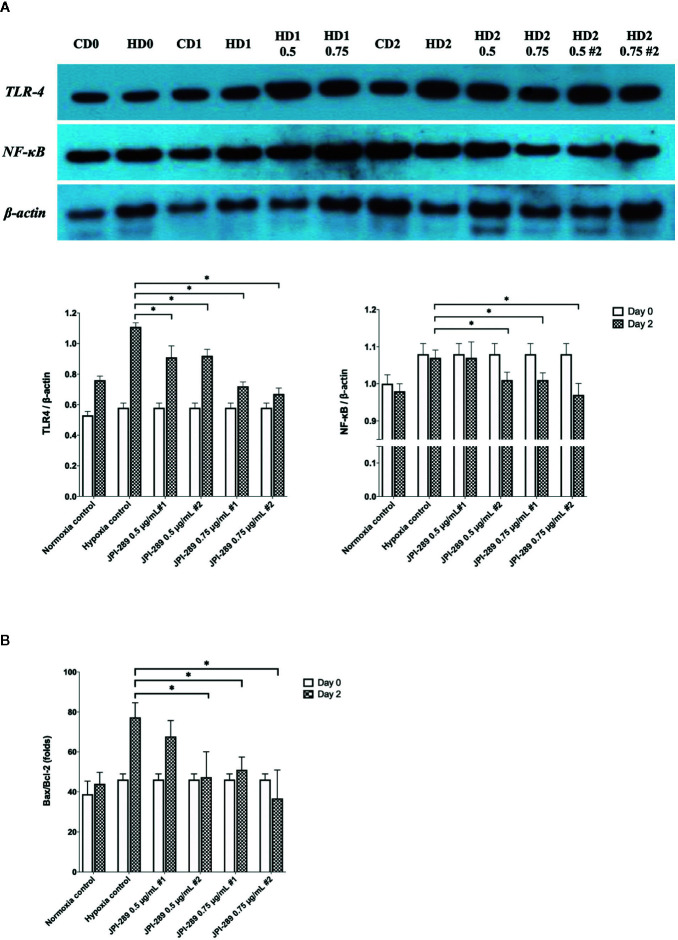
Analyses of cellular proinflammatory signaling pathways and apoptosis of hypoxic HK-2 cells. **(A)** Western blotting of TLR4 and NFκB showed that JPI-289 treatment reduced the expressions of TLR4 and NFκB compared with the hypoxia control group. Data are from six independent experiments. *P < 0.05, compared with the hypoxia control group. Statistical analysis was performed with the Mann-Whitney *U*-test. CD0, normoxia control group on day 0; CD1, normoxia control group on day 1; CD2, normoxia control group on day 2; HD0, the hypoxia group on day 0 in normoxia; HD1, the hypoxia group on day 1 in normoxia; HD2, the hypoxia group on day 2 in normoxia. The concentration of JPI-289 (ng/ml) is expressed in the parentheses. **(B)** Enzyme-linked immunosorbent assay of Bax and Bcl-2 showed that JPI-289 treatment reduced Bax/Bcl-2 ratios. Data are from six independent experiments. *P < 0.05, compared with the hypoxia control group. Statistical analysis was performed with the Mann-Whitney *U* test.

Considering that JPI-289 facilitated the proliferation of HK-2 cell after hypoxic insult, we investigated whether JPI-289 treatment would suppress proinflammatory signaling pathways in hypoxic HK-2 cells. Western blotting of TLR4 and NFκB in protein extracts of hypoxic HK-2 cells showed that 0.5 or 0.75 µg/ml JPI-289 reduced the expressions of TLR4 and NFκB compared with the hypoxia control group on day 2 after hypoxic insult (**Figure 7A**).

### JPI-289 Inhibited Apoptosis of Hypoxic HK-2 Cells

We also examined the apoptosis pathway in the hypoxic HK-2 cell model. ELISA of Bax and Bcl-2 proteins in protein extracts of HK-2 cells showed that JPI-289 treatment downregulated the expression of the proapoptotic protein Bax and upregulated the expression of the antiapoptotic protein Bcl-2 on day 2 after hypoxic insult (**Figure 7B**).

### JPI-289 Treatment Effectively Suppressed PARP-1 Activity

**Figure 8:**
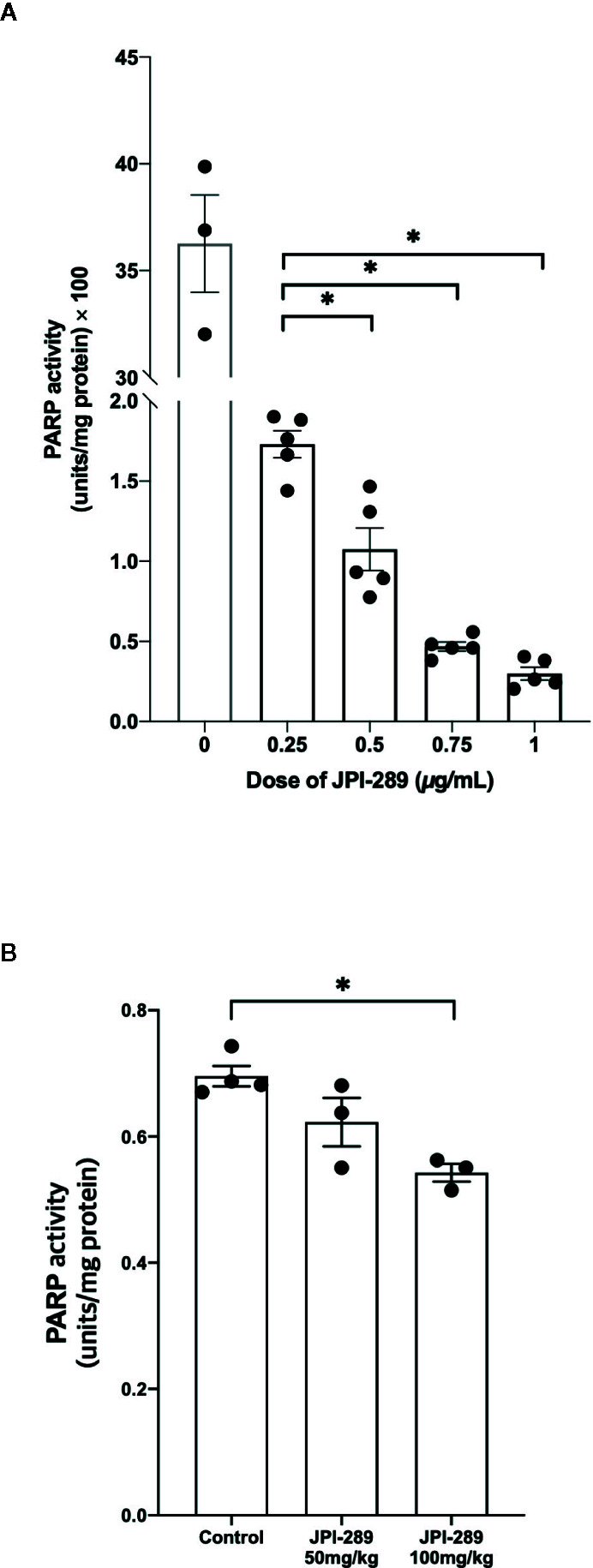
PARP-1 activities in the postischemic kidney protein extracts on day 3 after IRI. **(A)** JPI-289 effectively suppressed PARP-1 activity in a dose dependent fashion. Data are from two independent experiments. *P < 0.05, compared with the JPI-289 0.25 µg/ml treated group. Statistical analysis was performed with the Mann-Whitney *U* test. **(B)** Intrarenal PARP-1 activities in postischemic kidney protein extracts were lower in the JPI-289-treated mice. Data are from two independent experiments. *P < 0.05, compared with the control group. Statistical analysis was performed with the Mann-Whitney *U* test.

We measured PARP-1 activities depending on the JPI-289 dose applied in the hypoxic HK-2 cell model with biotinylated poly (ADP-ribose) loaded samples. The PARP-1 inhibitory effect was measured by the reduction in the amount of histone ribosylated by PARP-1 after JPI-289 treatment at the following concentrations: 0.25, 0.5, 0.75, and 1 µg/ml. PARP-1 activity was effectively inhibited by JPI-289 in a dose dependent fashion (**Figure 8A**).

To confirm that JPI-289 suppressed PARP-1 activity in the postischemic kidney, PARP-1 activities were measured in the postischemic kidney protein extracts. PARP-1 activities were lower in the postischemic kidney protein extracts of JPI-289-treated mice compared with controls in a dose-dependent manner (**Figure 8B**).

## Discussion

This study demonstrates that inhibition of PARP-1 by JPI-289 attenuated renal injury in a murine ischemic AKI model and facilitated the proliferation of hypoxic HK-2 cells. In addition, intrarenal inflammatory cell infiltration and proinflammatory cytokine/chemokine expressions were also mitigated by JPI-289 treatment. JPI-289 treatment during the early injury phase modified the proinflammatory micromilieu of postischemic kidneys to more favorable conditions with less inflammation and apoptosis without altering the proportion of intrarenal lymphocyte subpopulations, subsequently attenuating ongoing renal tissue damage. These results suggest the therapeutic potential of JPI-289 as a novel renoprotective agent without directly affecting adaptive immune system during the early injury phase of ischemic AKI.

Renal IRI is involved in various clinical situations including hypotension, shock from various etiologies, anesthesia, and surgical conditions such as cardiac and aorta surgery. Renal IRI is also an inevitable consequence of kidney procurement for kidney transplantation, which is associated with delayed graft function and rejections (1, 2, 7). Despite significant advances in immunosuppressive strategies and critical care medicine, conservative treatment including fluid therapy and dialysis remain the main treatment of ischemic AKI including DGF (1). Therefore, a novel pharmacological agent ameliorating renal IRI is required. Increased reactive oxygen and nitrogen radicals induce parthanatos, causing DNA fragmentation and pathological overactivation of PARP-1 in the postischemic kidney after IRI (7, 10). Since parthanatos plays a substantial role in the pathogenesis of renal IRI, this pathway may be a potential therapeutic target for the treatment of ischemic AKI (7).

Our results showed that JPI-289 exhibited immunomodulatory effects by suppressing intrarenal infiltration of total leukocytes and the production of proinflammatory cytokines/chemokines without changing the subpopulations of intrarenal lymphocytes. The trafficking of intrarenal total leukocytes was decreased in the postischemic kidneys of JPI-289-treated mice, but the proportions of total T cells, total B cells, and their subpopulations were comparable between the groups. In contrast, the infiltration of NK cells and macrophages was decreased in JPI-289-treated mice. Although the precise role of NK cell recruitment in renal IRI is not fully established (1), NK cells were reported to exert pathogenic effects in the development of AKI (18). The pathogenic roles of intrarenal macrophages during the injury phase after IRI were also well documented in previous studies (19, 20). JPI-289 treatment reduced the expression of intrarenal proinflammatory cytokines such as INF-γ, IL-2, and CCL2 that were reported to contribute to renal injury in postischemic kidneys (18, 21, 22). JPI-289 treatment also increased the expression of intrarenal VEGF, a well-known renoprotective chemokine after IRI (23, 24), whereas levels of IL-10, a well-known renoprotective cytokine (25), were comparable among groups. JPI-289 treatment also effectively suppressed the TLR4 and NFκB signaling pathways and apoptosis. Overall, these results show that JPI-289 treatment reduced DAMP signals in the postischemic kidney, suppressed intrarenal proinflammatory cascades, and subsequently switched the intrarenal immunologic micromilieu following IRI to favorable conditions mitigating renal injury.

We further investigated the effects of JPI-289 in a HK-2 cell hypoxia model and found that JPI-289 treatment reduced proinflammatory signaling pathways and facilitated the cellular proliferation of hypoxic HK-2 cells. This finding suggests that JPI-289 blocked hypoxia-mediated hyperactivation of PARP-1 and subsequently enhanced the proliferation of HK-2 cells even after hypoxic insult. These results are in agreement with previous *in vitro* studies demonstrating the effect of PARP-1 inhibitors on toxin-mediated HK-2 cell death (26) and ROS-mediated rat proximal tubular cell death (27).

Since robust inflammatory responses mediated by both innate and adaptive immune systems induce renal injury following IRI (1), we focused on intrarenal-infiltrated immune cells and the inflammatory micromilieu, highlighting the fact that PARP activation is involved in the development of proinflammatory cascades. Although several previous studies investigated the effects of other PARP inhibitors, such as benzamide and isoquinolone derivatives, in experimental kidney injury models, none of the studies assessed intrarenal immunologic micromilieu with reliable methods (11, 28–33). Unlike previously studied PARP inhibitors, JPI-289 is very potent and readily dissolves in saline or water. Its stability during long-term storage at room temperature and higher temperatures has been established in a previous study (12). In addition, as JPI-289 has high oral bioavailability, it could be used by oral administration in the clinical situation. JPI-289 has also shown promising results in ischemic stroke models (13) and pilot clinical trials are currently underway (NCT01983358).

There may be potential concerns with the involved mechanism of the PARP enzyme when considering the clinical application of PARP inhibitors in ischemic AKI. Inhibiting PARP-1 might increase the susceptibility to infection based on the immunostimulatory role of PARP-1. However, IRI following KT were known to enhance immunogenicity and increase the risk of allograft rejection (7). In our study, JPI-289 treatment reduced DAMP signals and suppressed overall proinflammatory cascades without changing the proportion of major effector cells of the adaptive immune system. Therefore, JPI-289 may be used as a novel drug to mitigate renal IRI without significant risk of serious infection. PARP inhibition also involves interference with DNA repair pathways, which may elicit concerns of developing malignancies. Although PARP inhibitors have been widely used in the clinical field as anticancer agents, secondary malignancies have been very rare and their causal relationship was weak (34). Furthermore, these risks may be even smaller because PARP blockade would be required only for a few days in the injury phase following IRI after KT or cardiovascular surgery.

There are several limitations in this study. First, the role of PARP in renal IRI has been only reported in animal models to date. The differences in the immune system between human and mice also limit the direct implication of our results to clinical settings. However, we also demonstrated reduced expression of proinflammatory signaling pathways and enhanced cellular proliferation by JPI-289 treatment after hypoxia in the human cellular AKI model using HK-2 cells. Second, the effects of JPI-289 on the postischemic kidney during the early injury phase following IRI were investigated in this study. Considering the important role of PARP1 in the DNA repair process, the remote effects of JPI-289 administered in the early injury phase on the postischemic kidney during repair phase need to be investigated. In addition, later time points of administration might result in different outcomes. Future studies to elucidate the most adequate time period for PARP inhibition are also required. Third, JPI-289 was intraperitoneally injected and tested at two different dosages in our study. As JPI-289 is orally available, the effects of oral administration with diverse dosages need to be investigated in future studies.

In conclusion, our study demonstrates that JPI-289 treatment suppressed the inflammatory response by reducing DAMP signals in postischemic kidneys and subsequently attenuated renal injury following IRI. These results indicate that early treatment of JPI-289 may be a novel therapeutic approach for ischemic AKI, especially for high risk patients of DGF. This study provides a rationale for the pharmacological use of PARP-1 inhibitors to reduce renal injury following IRI and accelerate recovery from ischemic AKI.

## Data Availability Statement

The raw data supporting the conclusions of this article will be made available by the authors, without undue reservation.

## Ethics Statement

The animal study was reviewed and approved by Samsung Medical Center Animal Care and Use Committee.

## Author Contributions

WH and HRJ designed the study. WH, HRJ, and KL performed experiments, analyzed and interpreted data, and drafted the manuscript. JJ, J-RK, JEL, GYK, Y-GK, DJK, and J-WK analyzed the data and revised the manuscript. All authors contributed to the article and approved the submitted version.

## Funding

This study was supported by the National Research Foundation of Korea (NRF-2017R1D1A1B04032172, NRF-2016R1A2B4008235, and NRF-2019R1A2B5B01069346).

## Conflict of Interest

The authors declare that the research was conducted in the absence of any commercial or financial relationships that could be construed as a potential conflict of interest.

The patent application for therapeutic use of JPI-289 in ischemic AKI was submitted by Samsung Medical Center and JEIL Pharmaceutical Co., LTD. to the Korean Intellectual Property Office.

All authors have no conflict of interest, financial, or otherwise with JEIL Pharmaceutical Co., LTD.

## Abbreviations

AKI, acute kidney injury; AU, arbitrary unit; BUN, blood urea nitrogen; CCL, CC-chemokine ligand; DAMP, damage-associated molecular pattern; DGF, delayed graft function; HK-2 cell, human kidney-2 cell; IFN-γ, interferon gamma; IL, interleukin; IRI, ischemia-reperfusion injury; KMNC, kidney mononuclear cell; KT, kidney transplantation; NAD^+^, nicotinamide adenine dinucleotide; NFκB, nuclear factor kappa-light-chain-enhancer of activated B cells; PARP, poly (ADP-ribose) polymerase; ROS, reactive oxygen species; SEM, standard error of mean; TLR4, toll-like receptor 4; TNF-α, tumor necrosis factor-α; VEGF, vascular endothelial growth factor.
