# Supplementary material for: Poly (ADP-Ribose) Polymerase Inhibitor Treatment as a Novel Therapy Attenuating Renal Ischemia-Reperfusion Injury
Source: Front Immunol. 2020 Oct 14;11:564288. doi: 10.3389/fimmu.2020.564288 (PMC7597449; doi:10.3389/fimmu.2020.564288)
Supplement: Supplementary file 1 [file Image_1.pdf]

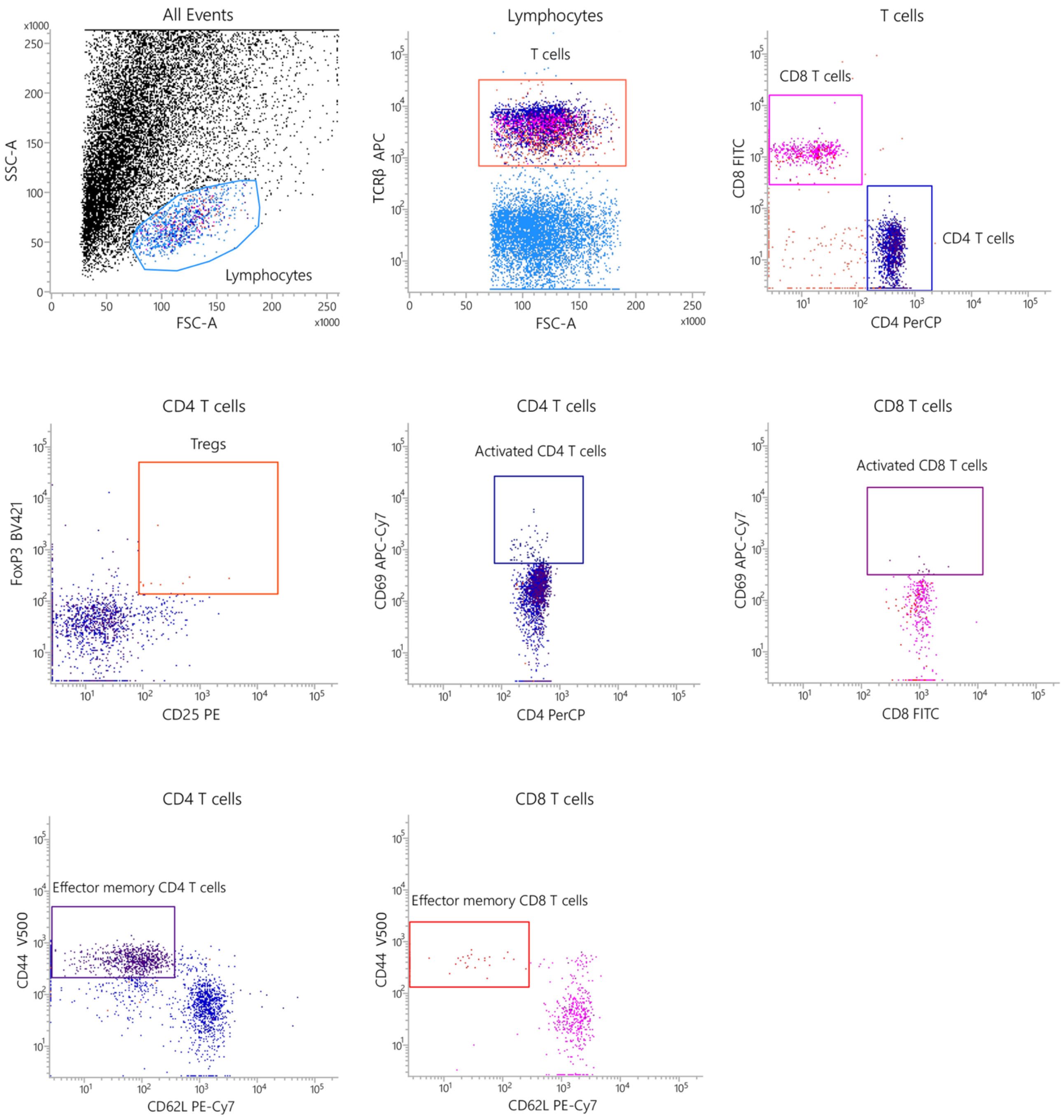

Supplemental Figure 1: Flow cytometry gating strategies for T cell subsets. KMNCs were stained with anti-TCRβ APC, anti-CD4 PerCP, anti-CD8 FITC, anti-CD25 PE, anti-FoxP3 BV421, anti-CD69 APC-Cy7, anti-CD44 V500, and anti-CD62L PE-Cy7. Lymphocytes were first identified with forward scatter (FSC) and side scatter (SSC) gates. $TCR\alpha\beta^+$ cells were gated and then analyzed for the expression of CD4 and CD8. Regulatory T cells were identified by the $CD25^+$ and $FoxP3^+$ gates within $CD4^+$ T cells. Activated CD4 T and CD8 T cells were identified by the $CD69^+$ gate within CD4 and CD8 T cells, respectively. Effector memory CD4 and CD8 T cells were identified by the $CD44^{high}$ and $CD62L^-$ gates within CD4 and CD8 T cells, respectively.

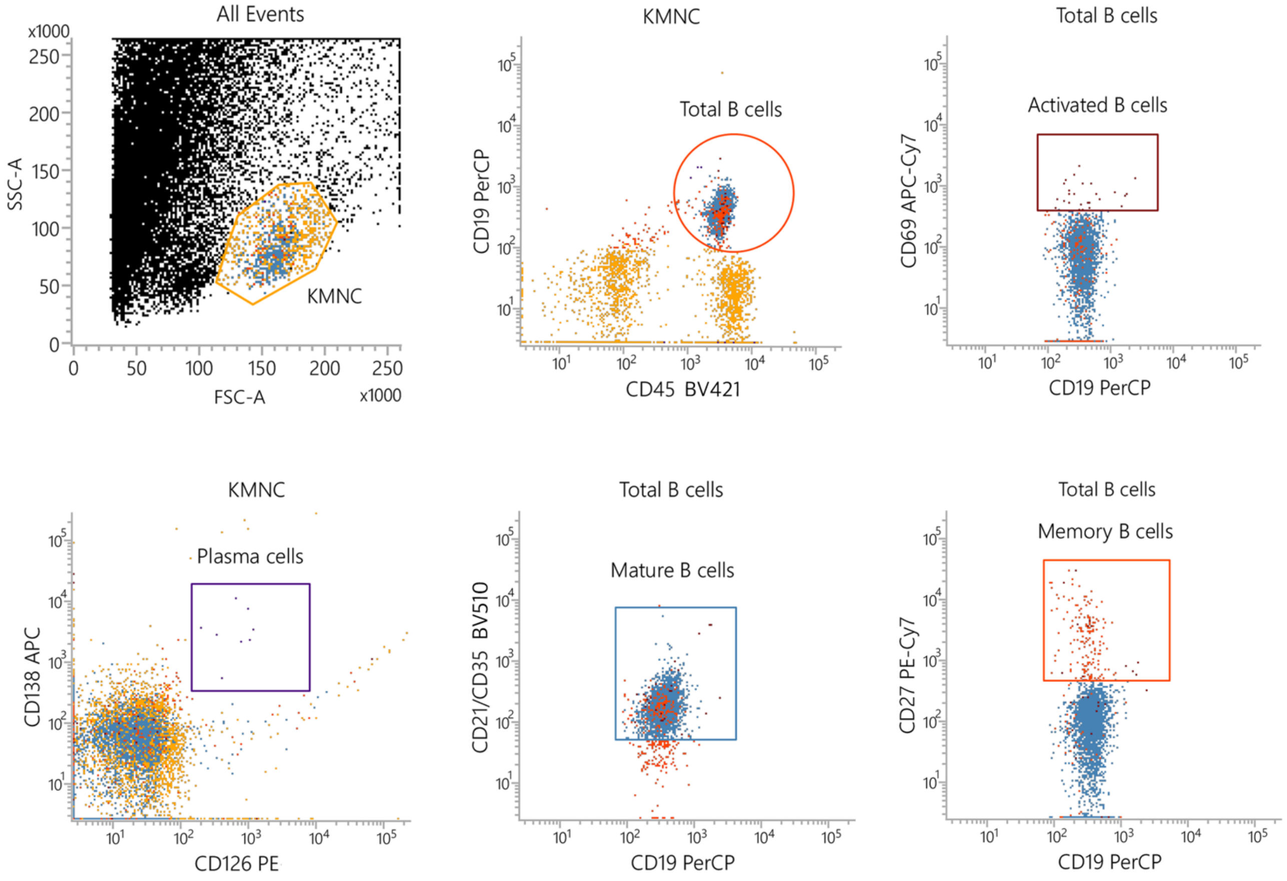

Supplemental Figure 2: Flow cytometry gating strategies for B cell subsets. KMNCs were stained with anti-CD45 BV421, anti-CD19 PerCP, anti-CD69 APC-Cy7, anti-CD126 PE, anti-CD138 APC, anti-CD21/35 BV510, and anti-CD27 PE-Cy7. Lymphocytes were first identified with FSC and SSC gates. Total B cells were identified by the CD45^{+} and CD19^{+} gates within the FSC and SSC based lymphocytes gate. Activated B cells were identified by the CD69^{+} gate within total B cells. Plasma cells were identified by the CD126^{+} and CD138^{+} gates within the FSC and SSC based lymphocytes gate. Mature B cells were identified by the CD21/35^{+} gate within total B cells. Memory B cells were identified by the CD27^{+} gate within total B cells.

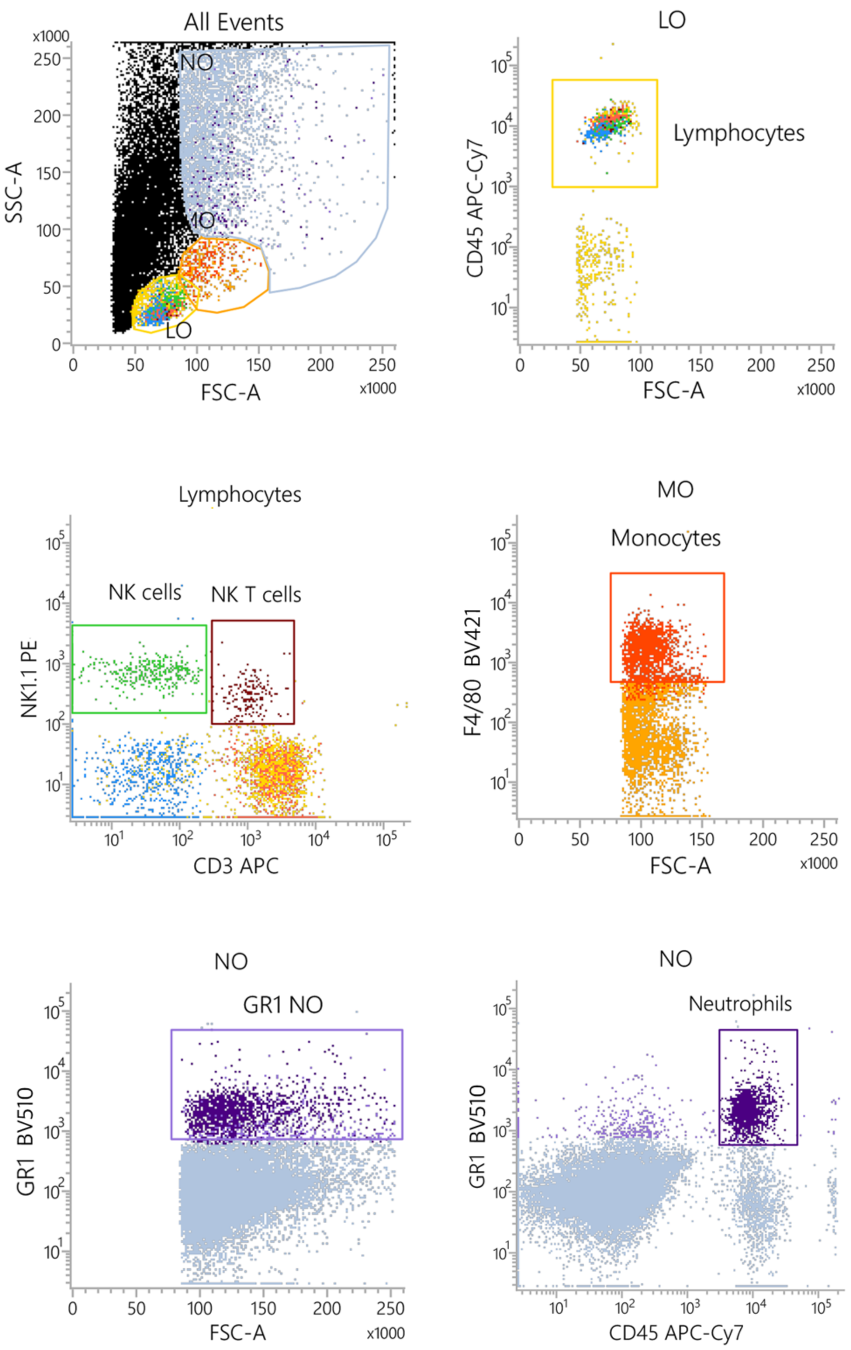

Supplemental Figure 3: Flow cytometry gating strategies for NK cells, NK T cells, macrophages, and neutrophils. KMNCs were stained with anti-CD45 APC-Cy7, anti-CD3 APC, anti-CD19 PerCP, anti-NK1.1 PE, anti-F4/80 BV421, and anti-GR1 BV510. Lymphocytes (yellow color), monocytes (orange color), and granulocytes (light blue color) were first identified based on their FSC and SSC. $CD45^+$ cells were gated to identify lymphocytes within FSC and SSC based lymphocytes population. NK T cells were identified by the $CD3^+$ and $NK1.1^+$ gates within the lymphocytes gate. NK cells were identified by the $CD3^-$ and $NK1.1^+$ gates within lymphocytes. Macrophages were identified by the $CD45^+$ and $F4/80^+$ gates within the FSC and SSC based monocyte population. Neutrophils were identified by the $CD45^+$ and $GR1^+$ gates within the FSC and SSC based granulocyte population.
